# Circ_FURIN knockdown assuages Testosterone-induced human ovarian granulosa-like tumor cell disorders by sponging miR-423-5p to reduce MTM1 expression in polycystic ovary syndrome

**DOI:** 10.1186/s12958-022-00891-9

**Published:** 2022-02-17

**Authors:** Xia Xu, Rui Guan, Ke Gong, Huaibing Xie, Lei Shi

**Affiliations:** 1 Department of Obstetrical, The Hospital of Bayannaoer, Bayannaoer City, Inner Mongolia China; 2 Department of Gynaecology, The Hospital of Bayannaoer, Bayannaoer City, Inner Mongolia China; 3 Department of Oncology, Huai’an Second People’s Hospital, Affiliated to Xuzhou Medical University, Qingjiangpu District, Huai’an City, No.62, Huaihai South Road, 223001 Jiangsu Province, China; 4 Department of Obstetrics and Gynecology, Hongze Huai’an District People’s Hospital, Hongze District, Huai’an City, No.102 Dongfeng Road, 223001 Jiangsu Province, China

**Keywords:** PCOS, Circ_furin, miR-423-5p, MTM1, TTR

## Abstract

**Background:**

Polycystic ovary syndrome (PCOS) is a common endocrine disorder among reproductive-age women. The mechanism by which circular RNA (circRNA) drives PCOS development remains unclear. Thus, the study is designed to explore the role of a novel circRNA, circ_FURIN, in the PCOS cell model and the underlying mechanism.

**Methods:**

PCOS cell model was established by treating human ovarian granulosa-like tumor cells (KGN) with Testosterone (TTR). RNA expressions of circ_FURIN, microRNA-423-5p (miR-423-5p) and myotubularin 1 (MTM1) were detected by quantitative real-time polymerase chain reaction (qRT-PCR). Protein expression was checked by Western blot. Cell proliferation was investigated by a 5-Ethynyl-29-deoxyuridine assay, 3-(4,5-Dimethylthazol-2-yl)-2,5-diphenyltetrazolium bromide (MTT) assay and flow cytometry analysis for cell cycle. Apoptotic cells were quantified by flow cytometry analysis for cell apoptosis. The interplay between miR-423-5p and circ_FURIN or MTM1 was identified by dual-luciferase reporter and RNA pull-down assays.

**Results:**

Circ_FURIN and MTM1 expressions were significantly upregulated, whereas miR-423-5p was downregulated in the ovarian cortex tissues of PCOS patients and TTR-treated KGN cells compared with controls. Circ_FURIN depletion relieved TTR-induced proliferation inhibition and apoptosis promotion. Besides, knockdown of miR-423-5p, a target miRNA of circ_FURIN, rescued circ_FURIN knockdown-mediated effects under TTR treatment. MiR-423-5p remitted TTR-induced cell disorders by binding to MTM1. Moreover, circ_FURIN modulated MTM1 expression through miR-423-5p.

**Conclusion:**

Circ_FURIN silencing protected against TTR-induced dysfunction by the miR-423-5p/MTM1 pathway in human ovarian granulosa-like tumor cells.

## Introduction

Polycystic ovary syndrome (PCOS) is a metabolic endocrinopathy that is the most leading cause for anovulatory infertility, featured by menstrual irregularity, hyperandrogenism and polycystic ovaries [1]. The heterogeneous syndrome with variable phenotypes is also one of the common causes of estrogen-dependent tumors. Besides, the endocrine disorder increases the risk of complications, such as insulin resistance, diabetes and cardiovascular diseases [2]. The abnormal development of follicular in follicular fluid is also considered a vital feature of PCOS [3]. A previous reference has expounded that granulosa cells can secrete the major components of follicular fluid [4]. Thus, an in-depth investigation of the molecular mechanism of PCOS development using granulosa cells can provide a direction for identifying the molecular target of the disease.

As a noncoding RNA, circular RNA (circRNA) is a product of the modulated back-spliced RNA. CircRNA is defined as an endogenous RNA with a closed continuous loop configuration, involving a covalent junction between 5’ cap and 3’ polyadenylation tail [5]. The specific structure can resist RNase, which makes circRNA more stable than linear RNA. Recently, circRNA has been known to be stable in the cytoplasm and blood [6]. CircRNA plays different biological roles like the regulator of RNA polymerase II and the sponge of microRNA (miRNA) in regulating gene expression [7]. Besides, limited evidence has confirmed the involvement of circRNA in disease development, such as cancers, neurological diseases, immune diseases and reproductive system diseases [8]. Circ_FURIN (circ_0036881) has been discovered to be most significantly upregulated among the 40 differently expressed circRNAs in PCOS [9], as revealed by unsupervised hierarchical clustering analysis; However, no data is showing the function of the circRNA in the pathogenesis of PCOS.

MiRNA, first discovered in 1993, is present in serum, semen, and follicular fluid [10]. The small RNA molecule regulates post-transcriptional gene expression through combination with mRNA, further inhibiting mRNA expression, blocking protein translation or both [11]. Some studies have showed that miRNAs participate in follicular development, oocyte maturation as well as ovulation, and the abnormal expression of miRNAs is usually involved in PCOS [12]. Considerable studies suggested that miR-423-5p aggravated lung cancer [13] and prostate cancer [14], but inhibited ovarian cancer [15] and glioblastoma [16]. In addition, Xie and his colleagues found that miR-423-5p might mediate ovulation-induced ovarian response [17]. But no study has focused on the role of miR-423-5p in modulating PCOS development.

This work predicted the target miRNA and mRNA of circ_FURIN through circbank and starbase online databases. We hypothesized that circ_FURIN potentially interacted with miR-423-5p and myotubularin 1 (MTM1). Thus, we analyzed circ_FURIN expression in PCOS patients, and explored its role in PCOS development using a PCOS cell model, which was established by treating human ovarian granulosa-like tumor cells (KGN) with Testosterone (TTR). Further, we investigated whether the circ_FURIN/miR-423-5p/MTM1 pathway was required for PCOS progression.

## Materials and methods

### Clinical samples

The patients with PCOS were recruited from the Hospital of Bayannaoer to collect ovarian cortex tissues (*N* = 21), and PCOS diagnosis was performed in accordance with the Rotterdam 2003 criteria [18]. Thirteen women who had similar age with the PCOS patients and had predictable regular menstrual cycles but had no history of PCOS were gathered to collect normal ovarian cortex tissues (*N* = 13; healthy control, HC) owing to tubal or male factor infertility. The tissues from PCOS patients were collected during the laparoscopic infertility investigation, and normal ovarian cortex tissues were obtained during diagnostic laparoscopy or laparoscopic disinfection for pelvic pain. All patients signed the written informed consent. These participants had not taken hormonal drugs within six months before participating in the study. The protocols conducted in this study were approved by the Ethics Committee of The Hospital of Bayannaoer.

### Cell culture and treatment

Human ovarian granulosa-like tumor cells (KGN) were provided by Otwo Biotech (Shenzhen, China), and cultured in Dulbecco’s modified Eagle’s medium (DMEM; HyClone, Logan, UT, USA) supplied with 10% fetal bovine serum (FBS; Biosun, Shanghai, China) and antibiotics (TCI; Shanghai, China) at 37˚C with 5% CO_2_. KGN cells were treated with 1 or 10 μM TTR for 24 h to analyze the expression of circ_FURIN. To explore the role of circ_FURIN in PCOS, we treated KGN cells using 10 μM TTR for the defined time to mimic the PCOS cell model according to the previous reference [19].

### Cell transfection

Ribobio Co., Ltd. (Guangzhou, China) synthesized the small hairpin RNA for circ_FURIN (sh-circ_FURIN, 5’-ACCAGTGTGCGAGGAAGGCTT-3’), the mimics of miR-423-5p (miR-423-5p, 5’-UGAGGGGCAGAGAGCGAGACUUU-3’), the inhibitors of miR-423-5p (anti-miR-423-5p, 5’-AAAGUCUCGCUCUCUGCCCCUCA-3’), and corresponding controls (sh-NC, 5’-CCTCTACCTGTCGCTGAGCTGTAAT-3’; miR-NC, 5’-UUUGUACUACACAAAAGUACUG-3’ and anti-miR-NC 5’-CAGUACUUUUGUGUAGUACAAA-3’). The plasmid upregulating MTM1 expression in KGN cells was achieved by introducing the coding sequence of MTM1 into the pcDNA 3.1( +) vector (pcDNA; YouBio, Changsha, China). According to the standard instructions of the FuGENE6 transfection reagent (Roche, Basel, Switzerland), KGN cells were transfected with shRNA, miRNA mimics, miRNA inhibitors and plasmids.

### Quantitative real-time polymerase chain reaction (qRT-PCR)

Multisource RNA isolation reagents (Tiangen, Beijing, China) were used to extract RNA from tissues and cells. RNA purity was identified using a UV-3100PC spectrophotometer. Reverse transcription of RNA was conducted inferring to the guidebook of cDNA synthesis reagents (Thermo Fisher, Waltham, MA, USA). Then, qRT-PCR mixtures (Thermo Fisher) were used to quantify gene expression with a StepOne qRT-PCR Machine (Thermo Fisher). RNA expression was analyzed by the 2^−∆∆Ct^ method with normalization to U6 or glyceraldehyde 3-phosphate dehydrogenase (GAPDH). The primer sequences were presented in Table 1.

**Table 1:** Primers sequences used for qRT-PCR

Name	Primers (5’-3’)	
circ_FURIN (hsa_circ_0036881)	Forward	GCATTGCTGGTTCTATTTA
	Reverse	TAGTGCGTATCGAGGACTT
FURIN	Forward	CGAGCCCAAAGACATCGGGAAAC
	Reverse	CAGTCATTAAACCCATCTGCGGAGTAG
MTM1	Forward	GGAACCCCAGGATCAAGCAA
	Reverse	GCGAAGGACTGGAAGGTGAA
miR-423-5p	Forward	GTATGATGAGGGGCAGAGAG
	Reverse	TGGTGTCGTGGAGTCG
GAPDH	Forward	GGAGCGAGATCCCTCCAAAAT
	Reverse	GGCTGTTGTCATACTTCTCATGG
U6	Forward	GCTTCGGCAGCACATATACTAA
	Reverse	AACGCTTCACGAATTTGCGT

### Subcellular fractionation

After discarding the culture medium, the cells were washed using phosphate buffer solution (PBS), and the culture plates were placed on ice. The following steps were carried out in accordance with the guidebook of PARIS™ Kit (Thermo Fisher). Briefly, the cells were exposed to Cell Disruption Buffer to obtain homogenous lysates. After centrifugation, the nuclear pellet and cytoplasmic fraction were separated and lysed, followed by RNA isolation. Eventually, circ_FURIN, U6 and GAPDH expressions were analyzed by qRT-PCR.

### Stability analysis of circ_FURIN

The assay involving circ_FURIN stability was performed using RNase R (Xiyuan Biotechnology, Shanghai, China) and Actinomycin D (Rechemscience, Shang, China). The isolated RNA was subjected to incubation with RNase R at 37˚C for 30 min. Actinomycin D (2 μg/mL) was mixed with DMEM and added into culture plates to block RNA synthesis, with dimethylsulfoxide as a negative control. Then, circ_FURIN and FURIN expressions were analyzed by qRT-PCR.

### 5-Ethynyl-29-deoxyuridine (EdU) assay

KGN cells were treated with TTR, sh-circ_FURIN, sh-NC, anti-miR-423-5p, anti-miR-NC, MTM1 and pcDNA alone or jointly, and allowed to grow in 9.6 cm petri dishes for 48 h. Then, DNA synthesis was analyzed after passaging the cells into 96-well plates with EdU-labeled DMEM following the instruction of an EdU staining kit (Ribobio). Images were captured with a confocal microscope (Olympus, Tokyo, Japan).

### 3-(4,5-Dimethylthazol-2-yl)-2,5-diphenyltetrazolium bromide (MTT) assay

The experiment regarding MTT (Beyotime, Shanghai, China) analyzed cell proliferation. In brief, after various treatments, the cells were cultured in 96-well plates for the defined time. The MTT reagent was added into each well before performing cell culture in an incubator lasting 2 h, after which dimethylsulfoxide was incubated with these cells. Finally, a microplate reader (REAGEN, Shenzhen, China) was used to analyze these samples.

### Western blot analysis

Total proteins in treated KGN cells and ovarian cortex tissues were extracted with a protein extraction reagent (AmyJet, Wuhan, China), separated by polyacrylamide gels (Thermo Fisher), and wet-transferred onto nitrocellulose membranes. The membranes were subjected to blockage using skimmed milk, and then immunoblotted using the following antibodies specific to proliferating cell nuclear antigen (PCNA; 1:50; Thermo Fisher), B-cell lymphoma-2 (Bcl-2; 1:50; Thermo Fisher), BCL2-associated x protein (Bax; 1:100; Thermo Fisher), cleaved poly(ADP-ribose) polymerase 1 (Cleaved PARP; 1:1000; Thermo Fisher) and GAPDH (as a reference protein) (1:10,000; Thermo Fisher), followed by incubation with secondary antibodies. Quantity One software was applied to analyze the relative protein expression.

### Flow cytometry analysis for cell cycle and apoptosis

KGN cells were treated with TTR, plasmids or oligonucleotides and allowed to grow in 12-well plates for 48. For the cell cycle assay, the cells were harvested and incubated with RNase A (Sigma, St. Louis, MO, USA) and propidium iodide (Solarbio, Beijing, China) for 30 min. Cell apoptotic rate was analyzed after incubating the cells with Annexin V-FITC (Solarbio) and PI in the dark. Flow cytometer (Thermo Fisher) equipped with CellQuest software was employed to analyze cell cycle and apoptosis.

### Dual-luciferase reporter assay

Online databases circbank (http://www.circbank.cn/index.html) and starbase (http://starbase.sysu.edu.cn/agoClipRNA.php?source=mRNA) were used to predict the complementary sites of miR-423-5p with circ_FURIN and MTM1. The wild-type and mutant reporter plasmids of circ_FURIN (circ_FURIN-WT and circ_FURIN-MUT) and MTM1 (MTM1-WT and MTM1-MUT) were achieved by introducing their sequences with or without the binding sites into the multiple cloning site of the pmirGLO vector (YouBio). Then, KGN cells were transfected with the above plasmids, miR-423-5p or miR-NC, according to the aforementioned methods. Dual-Lucy Assay Kit (Solarbio) was used to analyze the samples.

### RNA pull-down assay

The biotinylated linearized circ_FURIN-WT (Bio-circ_FURIN-WT), circ_FURIN-MUT (Bio-circ_FURIN-MUT), MTM1-WT (Bio-MTM1-WT), MTM1-MUT (Bio-MTM1-MUT) and control (Bio-NC) were provided by GenePharma Company (Shanghai, China), and then incubated with lysates from KGN cells, followed by incubating with streptavidin-coupled beads (Invitrogen, Carlsbad, CA, USA) lasting 4 h. At last, the co-precipitated RNA in complexes was subjected to qRT-PCR analysis.

### Statistical analysis

The data from three independent duplicate tests were analyzed using GraphPad Prism, and expressed as means ± standard deviations. The significant differences were compared by Spearman’s correlation test, Wilcoxon rank-sum test, Student’s *t*-tests or analysis of variance with Tukey’s test. *P* < 0.05 indicated statistical significance.

## Results

### Circ_FURIN expression was upregulated in ovarian cortex tissues of PCOS patients

**Fig. 1:**
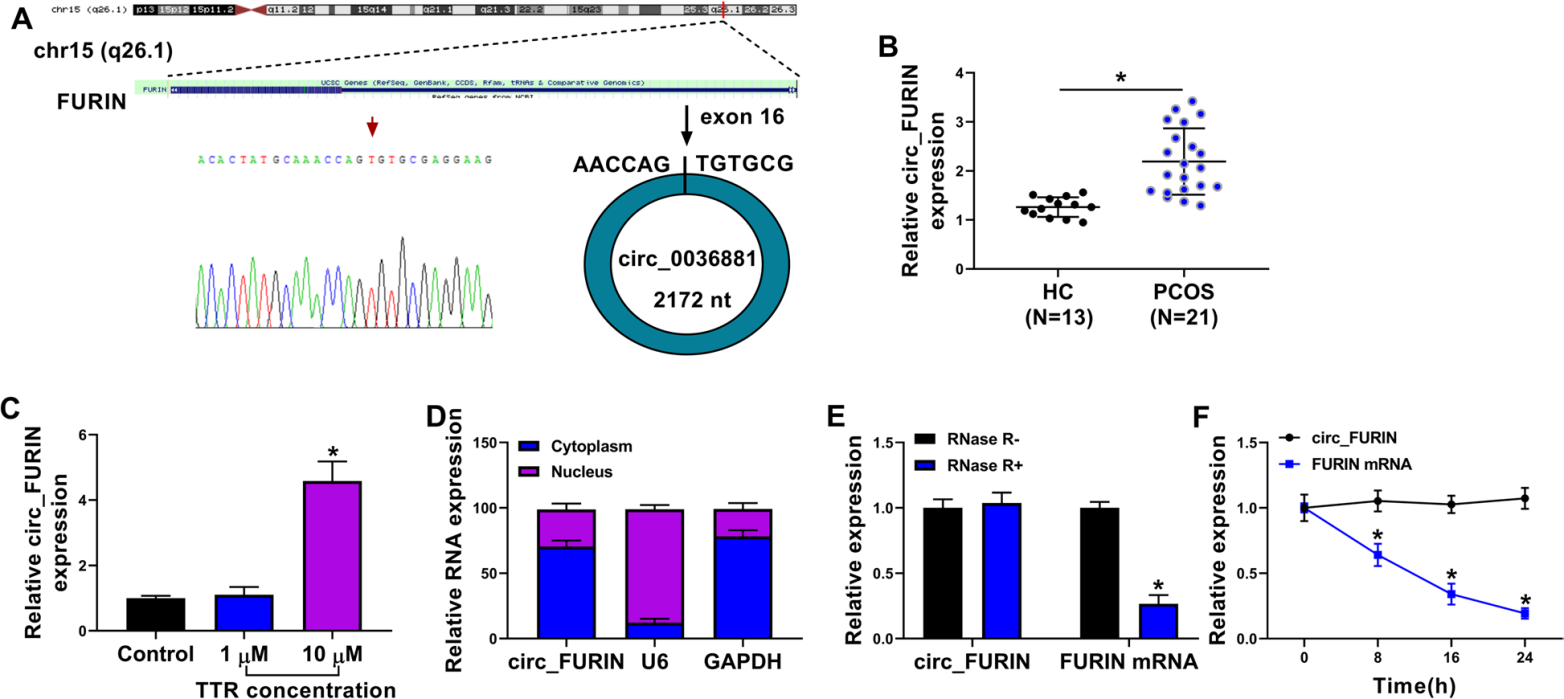
The high expression of circ_FURIN in ovarian cortex tissues of PCOS patients. **A** Circ_FURIN was generated via exon 16 circularization of the FURIN gene, and its presence was validated by qRT-PCR, followed by Sanger sequencing. **B** and **C** Circ_FURIN expression was detected by qRT-PCR in lesion ovarian cortex tissues of PCOS patients (*N* = 21), healthy ovarian cortex tissues (*N* = 13), mock KGN cells, and the KGN cells treated with TTR (1 and 10 μM). **D** Cytoplasmic and nuclear circ_FURIN analysis. **E** and **F** The stability of circ_FURIN was identified by RNase R and Actinomycin D treatment assays. Statistical significances were compared by Wilcoxon rank-sum test in (**B**), one-way analysis of variance with Tukey’s test in (**C**) and Student’s t-tests in (**E** and **F**). *P < 0.05

Circ_FURIN, also named circ_0036881, is looped and comprised of the exon 16 of FURIN gene, and its head-to-tail splicing was confirmed by Sanger sequencing (Fig. 1A). The expression pattern of circ_FURIN was first determined in the ovarian cortex tissues of PCOS patients. The data from Fig. 1B showed that the circ_FURIN expression was significantly increased in the lesion ovarian cortex tissues in comparison with healthy ovarian cortex tissues. Also, we found that circ_FURIN level was increased in TTR-treated KGN cells when compared with the untreated KGN cells, as illustrated in Fig. 1C. Subsequent data exhibited a higher expression of circ_FURIN in the cytoplasm as compared with its expression in the cell nuclei (Fig. 1D). Besides, our data identified the high stability of circ_FURIN. For instance, there was no significant difference in the circ_FURIN expression after RNase R treatment, but that of FURIN, the liner form of circ_FURIN, was significantly downregulated (Fig. 1E). Figure 1F exhibited that the transcript half-life of circ_FURIN exceeded 24 h, whereas that of FURIN was about 8 h. These results indicated that PCOS development might involve circ_FURIN.

### Circ_FURIN depletion remitted TTR-induced proliferation repression and apoptosis promotion

**Fig. 2:**
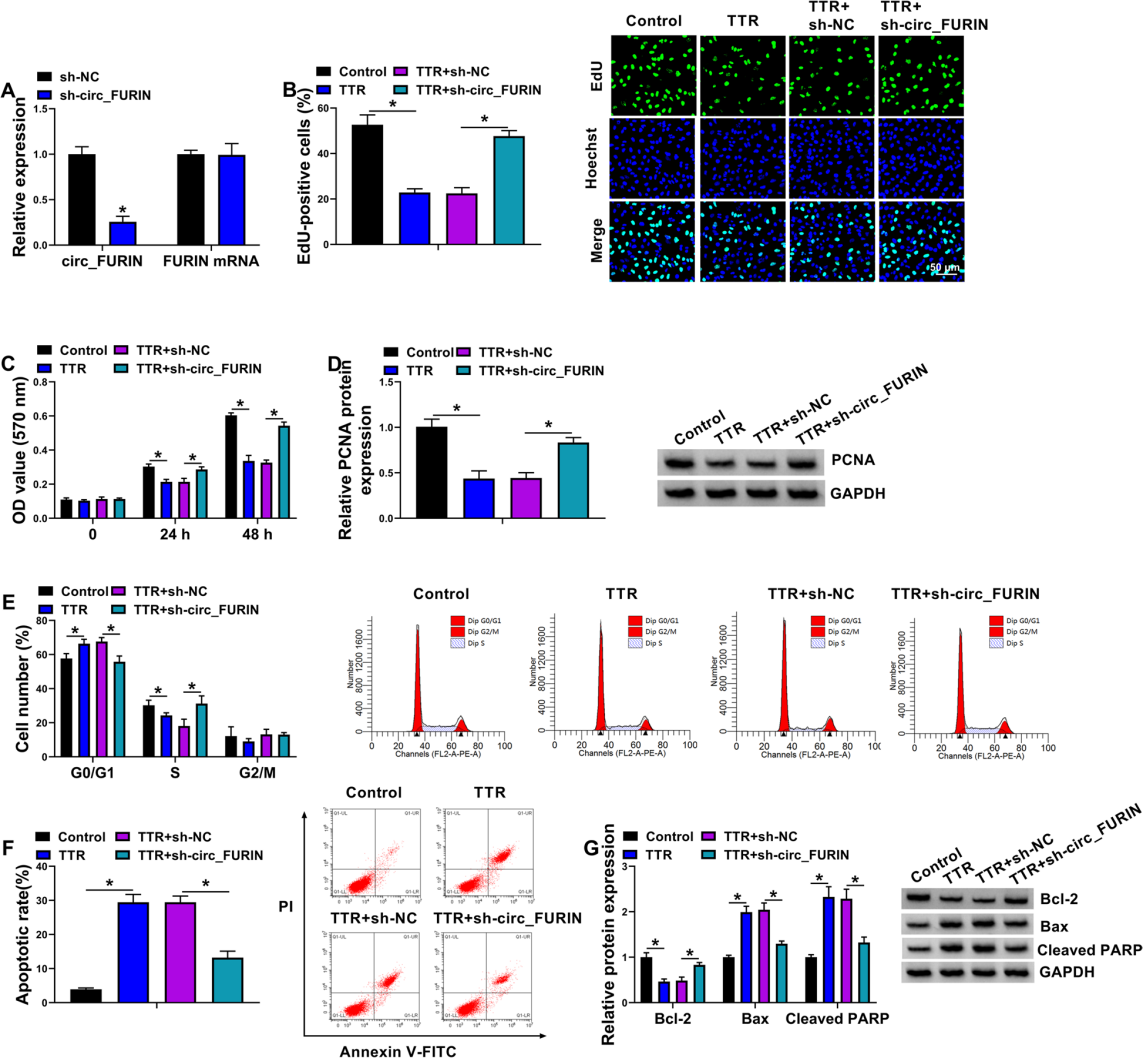
The effects of circ_FURIN knockdown on TTR-induced proliferation repression and apoptosis promotion. **A** The efficiency of circ_FURIN knockdown was determined by qRT-PCR in KGN cells. **B**-**G** KGN cells were treated with TTR, TTR + sh-NC, and TTR + sh-circ_FURIN, with mock KGN cells as a control, and cell proliferation was analyzed by EdU, MTT and flow cytometry analysis for cell cycle assays (**B**, **C** and **E**), the protein expressions of PCNA, Bcl-2, Bax and Cleaved PARP by Western blot (**D** and **G**), and cell apoptosis by flow cytometry analysis for cell apoptosis (**F**). Statistical significances were compared by Student’s t-tests in (**A**) and one-way analysis of variance with Tukey’s test in (**B**-**G**).*P < 0.05

The KGN cells were treated with TTR and the small hairpin RNA of circ_FURIN to determine the consequential effects on cell proliferation and apoptosis. The results from Fig. 2A showed that circ_FURIN expression was significantly decreased after transfection with sh-circ_FURIN, but there was no significant difference in FURIN expression, showing the high efficiency of sh-circ_FURIN in reducing the circ_FURIN expression. Subsequently, we found that TTR treatment inhibited DNA synthesis and cell proliferation, companied by a decrease of PCNA expression; however, these effects were attenuated after downregulation of circ_FURIN (Fig. 2B-D). Comparatively, TTR treatment induced cell arrest in the G0/G1 phase, which was relieved after circ_FURIN knockdown (Fig. 2E). Besides, TTR induced the apoptosis of KGN cells, accompanied by a decrease of Bcl-2 production and the increases of Bax and Cleaved PARP production; however, the combined treatment of TTR and sh-circ_FURIN rescued these effects (Fig. 2F-G). Collectively, these observations suggested that circ_FURIN silencing protected again TTR-induced KGN cell disorders.

### Circ_FURIN acted as a miR-423-5p sponge

**Fig. 3:**
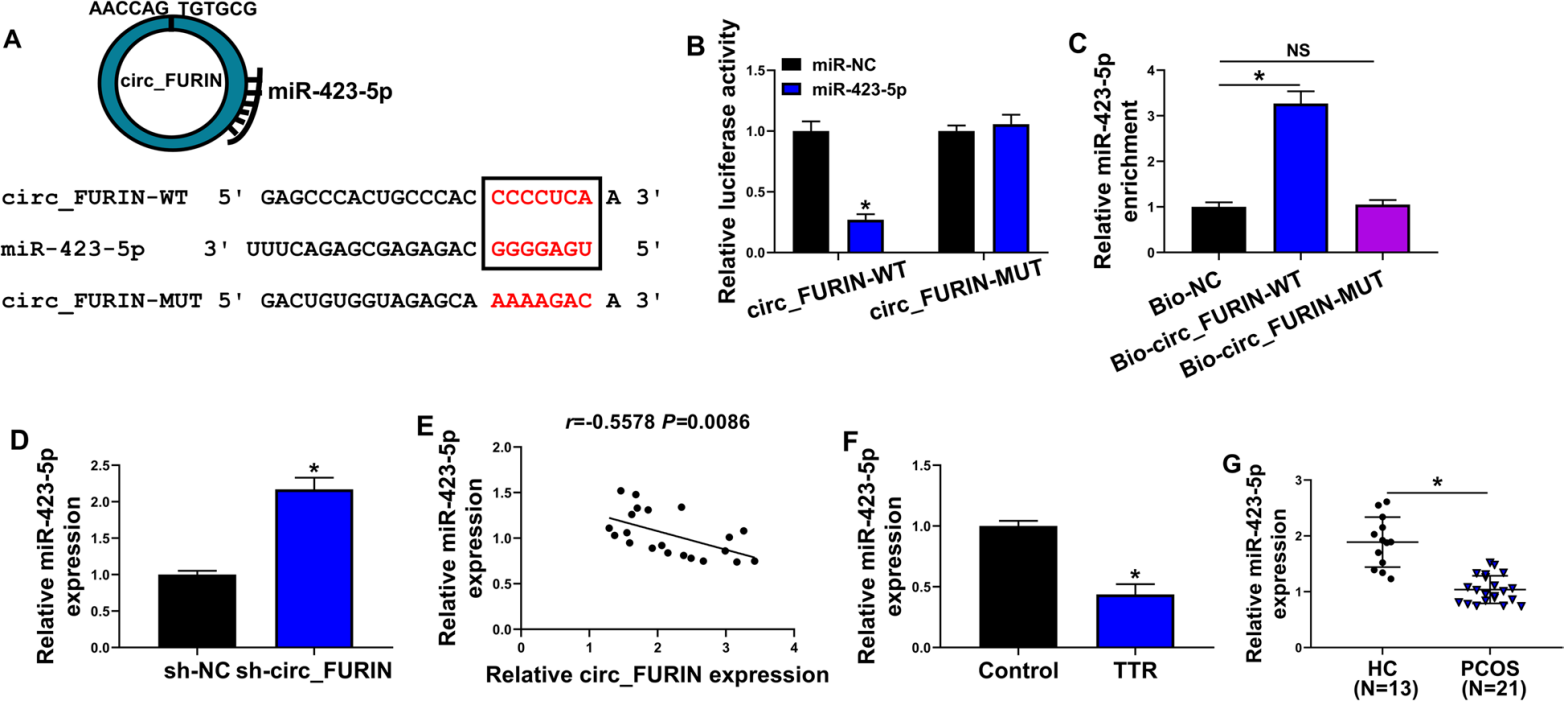
Circ_FURIN was associated with miR-423-5p. **A** The schematic illustration showing the complementary sites of circ_FURIN with miR-423-5p. **B** and **C** Dual-luciferase reporter and RNA pull-down assays were performed to analyze the interplay between circ_FURIN and miR-423-5p. **D** The effect of circ_FURIN depletion on miR-423-5p expression was determined by qRT-PCR. **E** Spearman correlation analysis was conducted to clarify the correlation of circ_FURIN and miR-423-5p in the ovarian cortex tissues of PCOS patients. **F** and **G** MiR-423-5p expression was detected by qRT-PCR in mock KGN cells, the KGN cells treated with TTR (10 μM), lesion ovarian cortex tissues of PCOS patients (*N* = 21) and healthy ovarian cortex tissues (*N* = 13). NS: no significant difference. Statistical significances were compared by Student’s t-tests in (**B**, **C**, **D** and **F**), Spearman’s correlation test in (**E**), and Wilcoxon rank-sum test in (**G**). *P < 0.05

The circbank online database was employed to predict the target miRNA of circ_FURIN. As presented in Fig. 3A, the seed sequence of miR-423-5p carried the binding sites of circ_FURIN. Also, the dual-luciferase reporter assay showed that the exogenetic expression of miR-423-5p significantly inhibited the luciferase activity of wild-type reporter plasmid of circ_FURIN, but not that of mutant (Fig. 3B). Meanwhile, biotinylated wild-type circ_FURIN dramatically enriched miR-423-5p compared with miR-423-5p expression in the Bio-NC or Bio-circ_FURIN-MUT (linearized circ_FURIN-MUT) group (Fig. 3C). The above results demonstrated that circ_FURIN combined with miR-423-5p. Then, we analyzed the association between miR-423-5p and circ_FURIN expressions. As exhibited in Fig. 3D-E, circ_FURIN silencing increased miR-423-5p, and circ_FURIN expression was negatively correlated with miR-423-5p expression in ovarian cortex tissues of PCOS patients. Consistently, miR-423-5p was significantly downregulated in TTR-stimulated KGN cells and ovarian cortex tissues of PCOS patients, compared with controls (Fig. 3F-G).

### Circ_FURIN regulated TTR-mediated KGN cell proliferation and apoptosis by targeting miR-423-5p

**Fig. 4:**
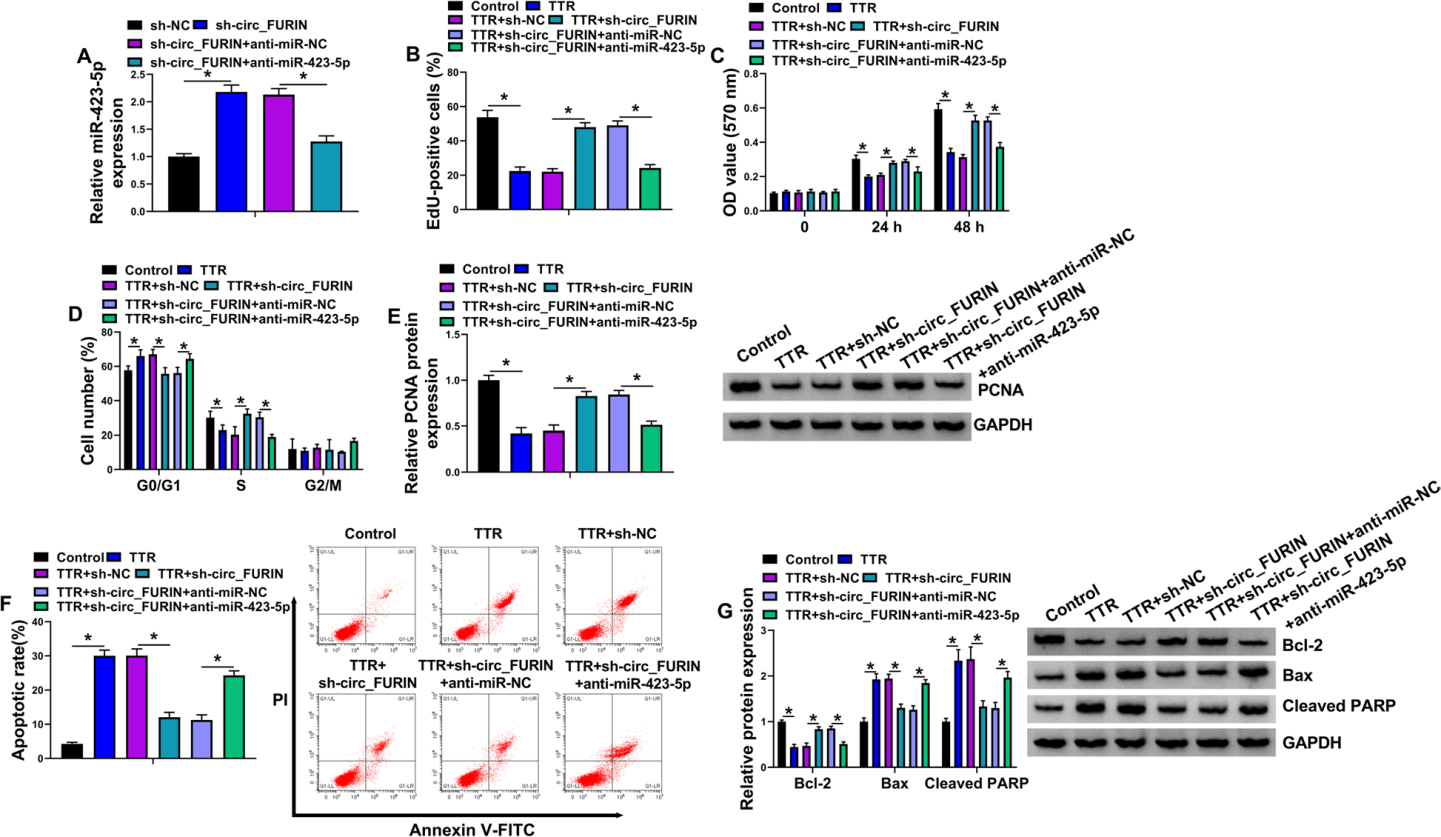
The effects between circ_FURIN and miR-423-5p on TTR-mediated proliferation and apoptosis. **A** qRT-PCR was used to determine the impacts between circ_FURIN knockdown and miR-423-5p depletion on the miR-423-5p expression. **B**-**G** KGN cells were treated with TTR, TTR + sh-NC, TTR + sh-circ_FURIN, TTR + sh-circ_FURIN + anti-miR-NC or TTR + sh-circ_FURIN + anti-miR-423-5p, with untreated KGN cells as a control, and cell proliferation was analyzed by EdU, MTT and flow cytometry analysis for cell cycle assays (**B**-**D**), the protein expressions of PCNA, Bcl-2, Bax and Cleaved PARP by Western blot (**E** and **G**), and cell apoptosis by flow cytometry analysis for cell apoptosis (**F**). Statistical significances were compared by one-way analysis of variance with Tukey’s test. *P < 0.05

Considering the binding relationship of circ_FURIN and miR-423-5p, we further analyzed whether miR-423-5p was involved in the regulation of circ_FURIN in TTR-stimulated KGN cells. The study first showed that the promoting effect of circ_FURIN knockdown on miR-423-5p expression was attenuated after miR-423-5p depletion (Fig. 4A). Subsequently, circ_FURIN knockdown attenuated TTR-reduced cell proliferation, which was remitted after transfection with miR-423-5p inhibitors (Fig. 4B-D). In support, the inhibitory impact of circ_FURIN depletion on TTR-induced downregulation of PCNA protein expression was rescued after a miR-423-5p absence (Fig. 4E). As expected, TTR-induced cell apoptosis and dysregulation of Bcl-2, Bax and Cleaved PARP were relieved by circ_FURIN depletion, whereas these effects were rescued after downregulation of miR-423-5p (Fig. 4F-G). Thus, these findings demonstrated that circ_FURIN regulated TTR-induced KGN cell dysfunction through miR-423-5p.

### Circ_FURIN modulated MTM1 expression through interaction with miR-423-5p

**Fig. 5:**
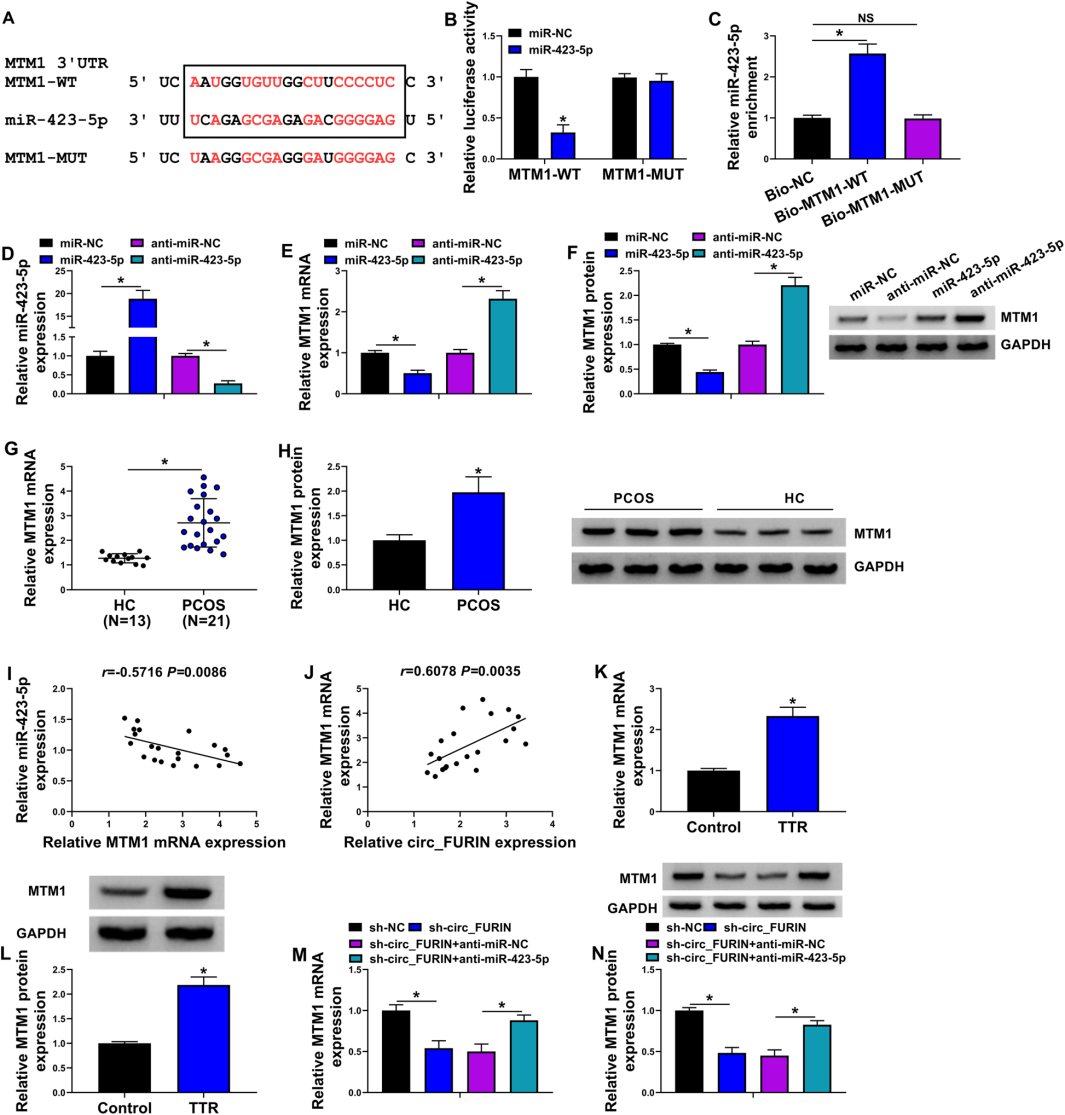
Circ_FURIN regulated MTM1 expression by associating with miR-423-5p. **A** The complementary sites of miR-423-5p with MTM1. **B** and **C** Dual-luciferase reporter and RNA pull-down assays were performed to identify the binding relationship of miR-423-5p and MTM1. **D** The efficiency of miR-423-5p overexpression and knockdown was analyzed by qRT-PCR. **E** and **F** The impacts of miR-423-5p overexpression and depletion on MTM1 expression were investigated by qRT-PCR and Western blot. **G** and **H** The mRNA and protein expressions of MTM1 were checked by qRT-PCR and Western blot, respectively, in the lesion ovarian cortex tissues of PCOS patients and healthy ovarian cortex tissues. **I** and **J** The linear correlation between MTM1 and miR-423-5p or circ_FURIN was analyzed by Spearman correlation analysis. **K** and **L** MTM1 expression was detected by qRT-PCR and Western blot in TTR-treated KGN cells and mock KGN cells. **M** and **N** The impacts between circ_FURIN knockdown and miR-423-5p depletion on MTM1 expression were investigated by qRT-PCR and Western blot. NS: no significant difference. Statistical significances were compared by Student’s t-tests in (**B**, **C**, **K** and **L**), one-way analysis of variance with Tukey’s test (**D**, **E**, **F**, **M** and **N**), Wilcoxon rank-sum test in (**G** and **H**), and Spearman’s correlation test in (**I** and **J**). *P < 0.05

The target gene of miR-423-5p was predicted by the starbase online database. As expected, MTM1, a candidate, contained the complementary sites of miR-423-5p (Fig. 5A), suggesting the potential of MTM1 as a target mRNA of miR-423-5p. Mechanism assays including dual-luciferase reporter and RIP assays were performed to examine the prediction. As shown in Fig. 5B, the luciferase activity of wild-type reporter plasmid of MTM1 was significantly inhibited after transfection with miR-423-5p mimics, but that of mutant had no response to miR-423-5p overexpression. Moreover, we found that miR-423-5p was dramatically enriched in the Bio-MTM1-WT group rather than the Bio-NC or Bio-MTM1-MUT group (Fig. 5C). The high efficiency of miR-423-5p overexpression or knockdown was presented in Fig. 5D. Then, the mRNA and protein expressions of MTM1 were downregulated after miR-423-5p introduction, but upregulated after miR-423-5p silencing (Fig. 5E-F). These findings implied that miR-423-5p bound to MTM1. Comparatively, lesion ovarian cortex tissues of PCOS patients exhibited the high expression of MTM1 (Fig. 5G-H). The Spearman correlation analysis showed that MTM1 expression was negatively correlated with miR-423-5p expression, but positively with circ_FURIN (Fig. 5I-J). The qRT-PCR analysis also showed the high expression of MTM1 in TTR-induced KGN cells (Fig. 5K-L). Further, the mRNA and protein expressions of MTM1 were inhibited after circ_FURIN knockdown, which was rescued by miR-423-5p downregulation (Fig. 5M-N). Collectively, these results manifested that circ_FURIN regulated MTM1 expression by associating with miR-423-5p.

### MiR-423-5p regulated TTR-induced KGN cell disorders through MTM1

**Fig. 6:**
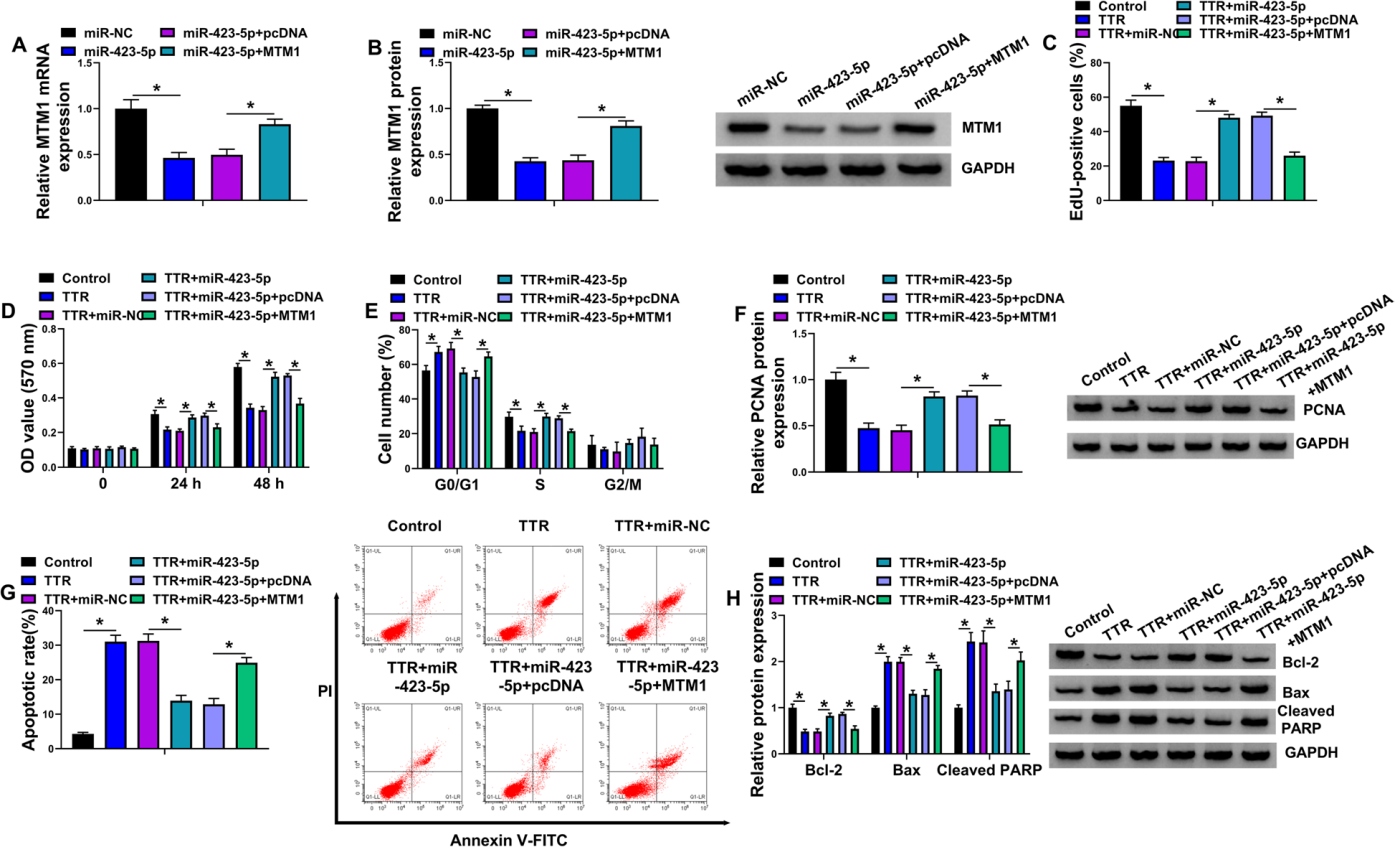
MiR-423-5p regulated TTR-induced KGN cell disorders through MTM1. **A** and **B** The effects between miR-423-5p and MTM1 on MTM1 expression were determined by qRT-PCR and Western blot analysis. **C**-**H** KGN cells were treated with TTR, TTR + miR-NC, TTR + miR-423-5p, TTR + miR-423-5p + pcDNA or TTR + miR-423-5p + MTM1 with mock KGN cells as a control, and cell proliferation was analyzed by EdU, MTT and flow cytometry analysis for cell cycle assays (**C**-**E**), the protein expressions of PCNA, Bcl-2, Bax and Cleaved PARP by Western blot (**F** and **H**), and cell apoptosis by flow cytometry analysis for cell apoptosis (**G**). Statistical significances were compared by one-way analysis of variance with Tukey’s test. *P < 0.05

MiR-423-5p-overexpressed cells were treated with MTM1 (the overexpression plasmid of MTM1) to explore whether MTM1 participated in the regulation of miR-423-5p in TTR-treated KGN cells. As analyzed by qRT-PCR and Western blot, the inhibitory impacts of miR-423-5p on MTM1 mRNA and protein expressions were attenuated after MTM1 overexpression (Fig. 6A-B). Then, the study found that the increased proliferation and PCNA expression induced by miR-423-5p upregulation was rescued after transfection with MTM1 (Fig. 6C-F). In contrast, miR-423-5p overexpression inhibited cell apoptosis and the production of Bax and Cleaved PARP, but promoted Bcl-2 expression in TTR-treated KGN cells; however, these effects were remitted by ectopic expression of MTM1 (Fig. 6G-H). In a word, these results demonstrated the involvement of MTM1 in miR-423-5p-mediated actions in TTR-treated KGN cells.

## Discussion

PCOS manifests as an ovulation disorder and is closely associated with the development of metabolic abnormalities, affecting more than five percent of women of reproductive age [20]. It has been found that hyperandrogenism mainly contributes to the PCOS occurrence [21]. On that account, we established the PCOS cell model by treating KGN cells with TTR to explore the role of circ_FURIN in the pathogenesis of PCOS. We found that circ_FURIN was highly expressed in PCOS patients, and circ_FURIN knockdown protected against TTR-induced KGN cell disorders. Besides, our data suggested that the underlying mechanism of TTR-induced KGN cell dysfunctions involved the circ_FURIN/miR-423-5p/MTM1 pathway.

A recent study indicated that circRNA participated in PCOS development. For example, loss of exosomal circRNA low density lipoprotein receptor (circ_LDLR) in follicle fluid inhibited estradiol production through sponging miR-1294 in PCOS [22]. An earlier study elucidated that circ_0023942 impeded granulosa cell proliferation, and the underlying mechanism might involve the MAPK signaling pathway, as predicted by GO and KEGG analysis [23]. In another study, circ_0118530 knockdown inhibited KGN cell activity and migration but induced cell apoptosis in a miR-136-dependent manner [24]. However, no study was performed to explore the role of circ_FURIN in PCOS development. In this work, circ_FURIN was identified to be augmented in PCOS patients and TTR-treated KGN cells. Loss of circ_FURIN assuaged TTR-induced proliferation inhibition and apoptosis promotion, which indicated that circ_FURIN might act as a promoter in PCOS progression. Besides, we proved that circ_FURIN targeted miR-423-5p.

Accumulating evidence has demonstrated that miRNA is expressed in different organs [25], playing vital parts in cell proliferation, apoptosis and differentiation. In particular, study data suggest the different expressions of miRNAs in PCOS patient, as compared with the patients without the disease [26]. Recently, an increasing number of research studies have exhibited that some miRNAs have a close association with PCOS development, thereby employed as new diagnostic markers for the illness [27, 28]. As reported, miR-409 improved the pregnancy rate in the PCOS rat model, and affected clinical phenotypes of the offspring of these rats [29]. MiR-99a inhibited the proliferation of granulosa cells, which were isolated from the follicular fluid of PCOS patients, but induced cell apoptosis through interaction with insulin-like growth factor 1 [30]. Enforced expression of miR-17-5p decelerated granulosa cell apoptosis and accelerated cell proliferation by regulating phosphatase and tensin homolog (PTEN) in PCOS [31]. In the present work, we found the low expression of miR-423-5p in PCOS patients, and a negative correlation between miR-423-5p and circ_FURIN. Besides, elevated expression of miR-423-5p in KGN cells assuaged TTR-induced cell disorders. Also, our data suggested that circ_FURIN mediated TTR-caused KGN cell dysfunction through miR-423-5p.

Myotubularins are a class of highly conserved proteins and are composed of 14 myotubularin members, regulating membrane trafficking, cell growth and cell autophagy [32]. As a member of myotubularins, the MTM1 gene consists of 15 exons and is necessary for skeletal muscle maintenance [33]. Considerable evidence has suggested that MTM1 plays an important role during intracellular membrane trafficking and vesicle transport due to its activity towards PtdIns3P and PtdIns(3,5)P2 [34, 35]. Also, Cao et al. described that MTM1 was associated with vacuolar protein sorting 15-vacuolar protein sorting 34 protein complex, which regulated protein sorting [36]. The above evidence demonstrated the importance of MTM1 in cell processes. Herein, we confirmed the involvement of MTM1 in PCOS, which was achieved by binding to miR-423-5p. We found that MTM1 was highly expressed in PCOS patients and TTR-stimulated KGN cells. MTM1 overexpression aggravated TTR-induced cell disorders. Besides, miR-423-5p modulated TTR-caused cell disorders through MTM1. Importantly, we demonstrated that circ_FURIN regulated MTM1 expression through MTM1.

However, some experiments need to be designed before translating these findings into clinical practice. Firstly, the rat model assay to verify the present data about the role of circ_FURIN during PCOS is lacking, which will be addressed using female Wistar rats. Secondly, our data lack direct evidence of MTM1 in mediating TTR-induced KGN cell disorders, which should be investigated in the future. In addition, it is necessary to validate the conclusion of the present study using granulosa cells isolated from patients with PCOS.

**Fig. 7:**
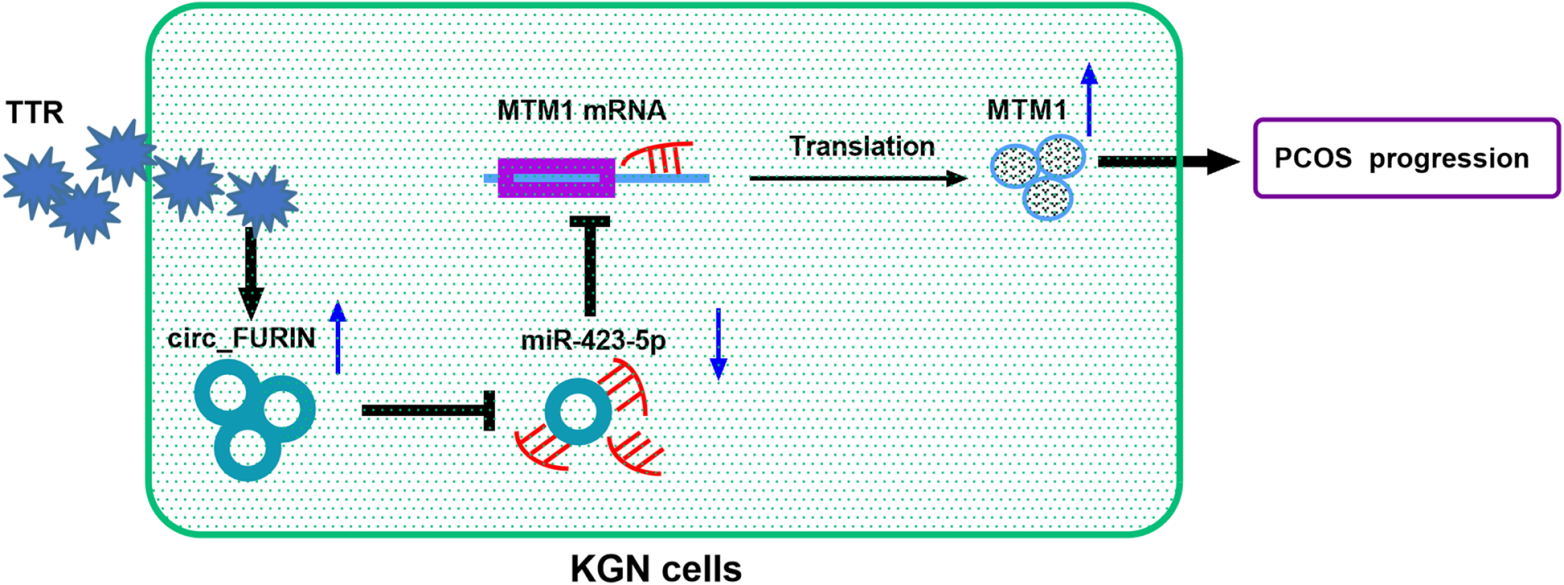
The mechanism underlying circ_FURIN regulating Testosterone-induced KGN cell disorders

Collectively, our work suggest that PCOS development may involve circ_FURIN. In mechanism, the high expression of circ_FURIN induces MTM1 production in a miR-423-5p-dependent manner, thereby inhibiting granulosa cell proliferation and promoting cell apoptosis (Fig. 7). The new finding demonstrates that circ_FURIN may be helpful for improving granulosa cell disorders in polycystic ovary syndrome.

## Data Availability

Please contact the correspondence author for the data request.
